# Association of Decreased Percentage of Vδ2^+^Vγ9^+^ γδ T Cells With Disease Severity in Multiple Sclerosis

**DOI:** 10.3389/fimmu.2018.00748

**Published:** 2018-04-10

**Authors:** Guzailiayi Maimaitijiang, Koji Shinoda, Yuri Nakamura, Katsuhisa Masaki, Takuya Matsushita, Noriko Isobe, Ryo Yamasaki, Yasunobu Yoshikai, Jun-ichi Kira

**Affiliations:** ^1^Department of Neurology, Neurological Institute, Graduate School of Medical Sciences, Kyushu University, Fukuoka, Japan; ^2^Division of Host Defense, Medical Institute of Bioregulation, Kyushu University, Fukuoka, Japan

**Keywords:** γδ T cell, Vδ2, Vγ9, regulatory CD4^+^ T, multiple sclerosis

## Abstract

We recently reported that deletion-type copy number variations of the T cell receptor (TCR) γ, α, and δ genes greatly enhanced susceptibility to multiple sclerosis (MS). However, the effect of abnormal TCR γδ gene rearrangement on MS pathogenesis remains unknown. In the present study, we aimed to clarify γδ TCR repertoire alterations and their relationship to clinical and immunological parameters in MS patients by comprehensive flow cytometric immunophenotyping. Peripheral blood mononuclear cells obtained from 30 untreated MS patients in remission and 23 age- and sex-matched healthy controls (HCs) were stained for surface markers and intracellular cytokines after stimulation with phorbol 12-myristate 13-acetate and ionomycin, and analyzed by flow cytometry. MS patients showed significantly decreased percentages of Vδ2^+^ and Vδ2^+^Vγ9^+^ cells in γδ T cells (*p*^corr^ = 0.0297 and *p*^corr^ = 0.0288, respectively) and elevated Vδ1/Vδ2 ratios compared with HCs (*p* = 0.0033). The percentages of interferon (IFN)-γ^+^Vδ2^+^ and interleukin (IL)-17A^+^IFN-γ^+^Vδ2^+^ cells in γδ T cells, as well as IFN-γ^+^ cells in Vδ2^+^ γδ T cells, were significantly lower in MS patients than in HCs (*p*^corr^ < 0.0009, *p*^corr^ = 0.0135, and *p*^corr^ = 0.0054, respectively). The percentages of Vδ2^+^ and Vδ2^+^Vγ9^+^ cells in γδ T cells were negatively correlated with both the Expanded Disability Status Scale score (*r* = −0.5006, *p* = 0.0048; and *r* = −0.5040, *p* = 0.0045, respectively) and Multiple Sclerosis Severity Score (*r* = –0.4682, *p* = 0.0091; and *r* = –0.4706, *p* = 0.0087, respectively), but not with age at disease onset, disease duration, or annualized relapse rate. In HCs, the percentages of Vδ2^+^ and Vδ2^+^Vγ9^+^ cells of total CD3^+^ T cells had strong positive correlations with the percentage of CD25^+^CD127^low/−^ cells in CD4^+^ T cells (*r* = 0.7826, *p* < 0.0001; and *r* = 0.7848, *p* < 0.0001, respectively), whereas such correlations were totally absent in MS patients. These findings suggest that decreased Vδ2^+^Vγ9^+^ γδ T cells are associated with disability in MS. Therefore, the Vδ1/Vδ2 ratio might be a candidate biomarker for predicting disease severity in MS.

## Introduction

Multiple sclerosis (MS) is an inflammatory demyelinating disease of the central nervous system (CNS) mediated by autoreactive T cells (1). Among T cells bearing T cell receptor (TCR) α and β chains (αβ T cells), interferon (IFN)-γ-secreting helper T (Th) 1 cells and interleukin (IL)-17-secreting Th17 cells play pathogenic roles in experimental autoimmune encephalitis (EAE), an animal model of MS (2, 3). In MS, Th1 and Th17 cells are increased at relapse in peripheral blood and suppressed by IFN-β treatment (4). These observations collectively suggest the involvement of Th1/Th17 cells in relapse induction. However, the percentage of Th1/Th17 cells in peripheral blood has no or a poor correlation with MS severity. Thus, surrogate immunological markers for disease severity present in peripheral blood remain to be established in MS.

γδ T cells express TCR γ and δ chains and are a distinct lineage of cells from αβ T cells, comprising 1–5% of lymphocytes in peripheral blood (5). These unconventional T cells respond quickly to specific pathogen-associated molecular patterns (6–8). Thus, γδ T cells play an important role in the early phase of host defense against bacteria (6), mycobacteria (7), and fungi (8). γδ T cells expressing various effector and regulatory molecules produce a variety of pro- and anti-inflammatory cytokines (9–11). As a result, these cells are involved in the pathogenesis of various inflammatory diseases (12). For example, γδ T cells infiltrate CNS tissues at the earliest phase of EAE. By innately producing IL-17 upon stimulation with IL-1β and IL-23, γδ T cells amplify Th17 autoimmune responses (13).

In MS, total γδ T cells are enriched in both the blood and cerebrospinal fluid while a fraction of CD161^high^CCR6^+^ γδ T cells are increased in the cerebrospinal fluid at relapse (14). Notably, γδ T cells are reported to be abundantly present in chronic active brain lesions (15–18). However, such γδ T cells possess limited diversity (18). Importantly, γδ T cells can lyse human brain-derived oligodendrocytes *via* the recognition of heat shock proteins (18, 19). We recently reported findings of a genome-wide copy number variation (CNV) association study where deletion-type CNVs at *TCRα* and γ loci greatly enhanced susceptibility to MS (20). Given that deletion-type CNV at the *TCRα* locus also covers *TCRδ* genes (5), we hypothesized that a deviation in *TCR*γδ gene rearrangement contributes to the pathogenesis of MS. Only two previous reports have described an increase of peripheral blood Vδ1^+^ γδ T cells in Caucasian MS patients (21, 22); however, neither study directly measured Vδ2^+^ γδ T cells. No previous studies have reported the peripheral blood Vδ and Vγ repertoires in MS, nor their relationship with αβ T cells. Therefore, the present study investigated alterations of γδ T cell subsets in the context of the TCR γδ repertoire in untreated MS patients by comprehensive flow cytometric immunophenotyping. Furthermore, we aimed to clarify the relationship between γδ T cell alterations and clinico-immunological parameters.

## Materials and Methods

### Study Participants

Study participants were 30 untreated MS patients and 23 healthy controls (HCs) (Table 1). All patients were thoroughly examined and regularly followed-up at a single MS center in Kyushu University Hospital. The diagnosis of MS was based on established criteria (23). MS patients, who were in remission, negative for anti-aquaporin 4 antibodies (24, 25), and not receiving corticosteroids or any disease-modifying therapies (DMTs) at least 6 months prior to the immunophenotyping, were enrolled between March 1 2016 and February 28 2017. The frequency of females and age at examination did not significantly differ between the two study groups. The study was approved by the Ethical Committee of Kyushu University, and conducted with written informed consent from all participants in accordance with the World Medical Association Declaration of Helsinki.

**Table 1:** Demographics of study participants.

	MS (*n* = 30)	HCs (*n* = 23)	*p* Value
Female, *n* (%)	27 (90.0)	17 (73.9)	NS
Age at examination, years	49.53 ± 14.09	43.48 ± 6.83	NS
Age at disease onset, years	32.50 ± 12.56	NA	NA
Disease duration, years	17.04 ± 12.17	NA	NA
Relapsing-remitting MS, *n* (%)	24 (80)	NA	NA
EDSS score	2.95 ± 2.65	NA	NA
MSSS	3.24 ± 3.11	NA	NA
Annualized relapse rate	0.31 ± 0.59	NA	NA
Prior history of DMTs, *n* (%)	5 (16.7)^†^	NA	NA
Prior history of corticosteroid, *n* (%)	9 (30.0)	NA	NA
Prior history of immunosuppressant, *n* (%)	2 (6.7)^††^	NA	NA

*Data are presented as the number (percentage) or mean ± SD*.

*^†^Five patients had a history of interferon β (*n* = 4) or fingolimod (*n* = 1) therapy*.

*^††^Two patients had a history of cyclosporine-A and azathioprine therapy, respectively*.

*DMTs, disease-modifying therapies; EDSS, Expanded Disability Status Scale; HCs, healthy controls; MS, multiple sclerosis; MSSS, Multiple Sclerosis Severity Score; NA, not applicable; NS, not significant*.

### Antibodies and Flow Cytometric Analysis

Peripheral blood mononuclear cells were collected by density gradient centrifugation using Lymphoprep tubes (AXIS-SHIELD Poc AS, Oslo, Norway) containing Ficoll-Paque (GE Healthcare, Little Chalfont, UK) and then suspended in RPMI-1640 medium (Wako, Osaka, Japan) supplemented with 10% fetal bovine serum (Gibco, Waltham, MA, USA). Immunophenotyping was performed using the antibodies shown in Tables S1 and S2 in Supplementary Material. For surface staining, cell suspensions were incubated with an optimal concentration of antibodies for 20 min at 4°C in the dark. For intracellular staining, cell suspensions were incubated with 25 ng/ml of phorbol 12-myristate 13-acetate (PMA; Sigma-Aldrich, St. Louis, MO, USA) and 1 µg/ml of ionomycin (Sigma-Aldrich) in the presence of 10 µg/ml of brefeldin A (Sigma-Aldrich) for 4 h at 37°C. Stained cells were analyzed on a FACSVerse flow cytometer (BD Biosciences, Franklin Lakes, NJ, USA).

γδ T cells (CD3^+^TCRγδ^+^TCRαβ^−^) were characterized by surface staining with anti-Vδ1, Vδ2, and Vγ9 antibodies, and then cytokine production was determined by intracellular cytokine staining for IL-17A and IFN-γ (Figures S1A,B in Supplementary Material). αβ T cells were characterized as CD4^+^ or CD8^+^ T cells, and subsequently as naïve T (Tnaïve, CCR7^+^CD45RA^+^), central memory T (Tcm, CCR7^+^CD45RA^−^), effector memory T (Tem, CCR7^−^CD45RA^−^), effector T (Teff, CCR7^+^CD45RA^−^), or activated T (HLA-DR^+^) cells by surface staining (Figure S2A in Supplementary Material). Regulatory CD4^+^ T (Treg) cells were defined as CD25^+^CD127^low/−^. In addition, CD4^+^CD25^+^CD127^low/−^ T cells expressing FoxP3 were measured in HCs (Figure S3 in Supplementary Material), because FoxP3 expression in CD4^+^CD25^+^ T cells has been reported to be tightly linked to the suppressive functions of Treg cells (26, 27). We found that FoxP3^+^ cells were 77.7 ± 7.9% of CD4^+^CD25^+^CD127^low/−^ T cells, and that the percentage of CD25^+^CD127^low/−^ T cells (defined as Tregs) had a significant positive correlation with CD25^+^FoxP3^+^ T cells in CD4^+^ T cells (*r* = 0.6396, *p* = 0.0138) (Figure S4 in Supplementary Material). These data are consistent with previous studies (28, 29).

For intracellular cytokine staining of αβ T cells after *in vitro* stimulation with PMA and ionomycin, IL-17A, IFN-γ, IL-4, and granulocyte-macrophage colony-stimulating factor (GM-CSF) were measured in CD4^+^ T cells, while IL-17A and IFN-γ were measured in CD8^+^ T cells (Figure S2B in Supplementary Material). B cells (CD19^+^CD3^−^) were characterized by surface staining as class-switched memory (CS^+^ memory, CD27^+^IgD^−^), non-class-switched memory (CS^−^ memory, CD27^+^IgD^+^), naïve B (CD27^−^IgD^−^), and transitional B (CD24^+^CD38^+^) cells and plasmablasts (CD38^high^CD20^−^) (Figure S5 in Supplementary Material). Appropriate isotype controls were used in each experiment. The data were analyzed using FlowJo software (TreeStar, San Carlos, CA, USA).

### Statistical Analysis

Fisher’s exact test was used to compare categorical variables, and the Wilcoxon rank sum test was used to analyze continuous scales. Correlations among continuous scales were calculated using Spearman’s rank correlation coefficient. Uncorrected *p* values (*p*^uncorr^) were multiplied by the number of comparisons to calculate the corrected *p* values (*p*^corr^), as indicated in the footnote of the tables (Bonferroni–Dunn’s correction). Statistical analysis was performed using JMP Pro 12.2.0 software (SAS Institute, Cary, NC, USA). A *p*-value <0.05 was considered statistically significant.

## Results

### Distinct Repertoire of γδ T Cells in MS Patients

**Figure 1:**
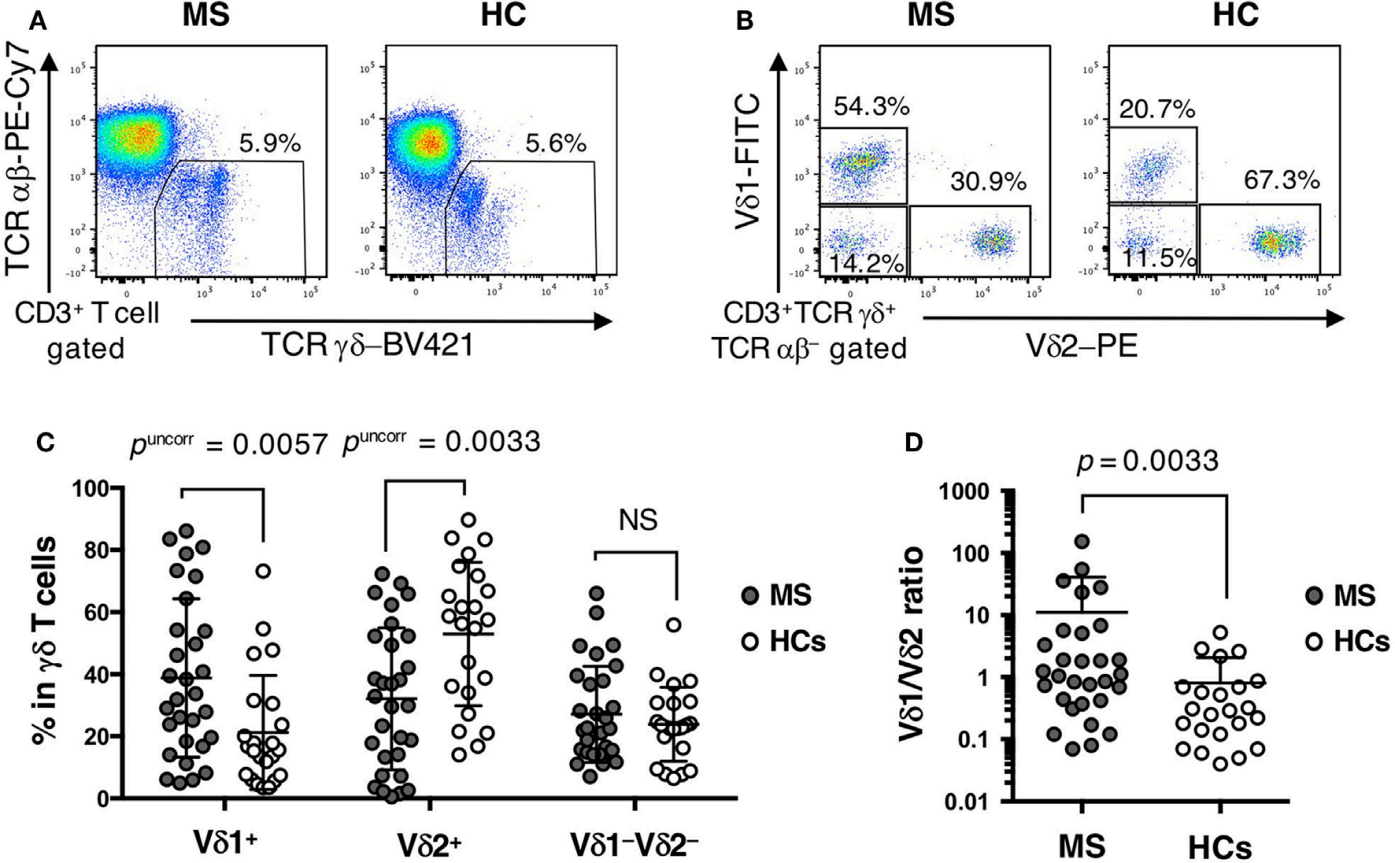
Distinct repertoire of γδ T cells between MS patients and HCs. **(A)** Representative examples of flow cytometric analyses for αβ and γδ T cells in MS patients and HCs. **(B)** Representative examples of flow cytometric analyses for Vδ1^+^, Vδ2^+^, and Vδ1^−^Vδ2^−^ cells in γδ T cells in MS patients and HCs. **(C)** The frequencies of Vδ1^+^, Vδ2^+^, and Vδ1^−^Vδ2^−^ cells in γδ T cells. **(D)** The Vδ1/Vδ2 ratio in MS patients and HCs. Closed circles represent MS patients, while open circles indicate HCs. Abbreviations: MS, multiple sclerosis; HCs, healthy controls.

The percentage of total γδ T cells (TCRγδ^+^TCRαβ^−^) in CD3^+^ T cells did not differ significantly between MS patients and HCs (Table 2; Figure 1A). However, within γδ T cells, the percentages of Vδ2^+^, Vδ2^+^Vγ9^+^, and Vδ1^−^Vδ2^−^Vγ9^+^ cells were decreased (Vδ2^+^: *p*^corr^ = 0.0297; Vδ2^+^Vγ9^+^: *p*^corr^ = 0.0288; and Vδ1^−^Vδ2^−^Vγ9^+^: *p*^corr^ = 0.0882) in MS patients compared with HCs. By contrast, the increase of Vδ1^+^, Vδ1^+^Vγ9^+^, and Vδ1^+^Vγ9^−^ cells in MS patients was not significant after Bonferroni–Dunn’s correction (Vδ1^+^: *p*^corr^ = 0.0513; Vδ1^+^Vγ9^+^: *p*^corr^ = 0.1323; and Vδ1^+^Vγ9^−^: *p*^corr^ = 0.0792) (Figures 1B,C). Moreover, the percentages of Vδ2^+^ and Vδ2^+^Vγ9^+^ γδ T cells in CD3^+^ T cells were significantly reduced in MS patients compared with HCs, even after Bonferroni–Dunn’s correction (Vδ2^+^: *p*^corr^ = 0.0380; and Vδ2^+^Vγ9^+^: *p*^corr^ = 0.0340). These results suggest that the reduction of Vδ2^+^ γδ T cells, mostly composed of Vδ2^+^Vγ9^+^ γδ cells, was the primary difference between MS patients and HCs. We also examined the ratio of Vδ1^+^ to Vδ2^+^ γδ T cells (Vδ1/Vδ2 ratio) and found that MS patients had a significantly higher Vδ1/Vδ2 ratio than HCs (mean ± SD, 11.05 ± 29.56 vs. 0.80 ± 1.26, *p* = 0.0033) (Figure 1D).

**Table 2:** Comparison of γδ T cell subpopulations between MS patients in remission and HCs.

	MS (*n* = 30)	HCs (*n* = 23)	*p*^uncorr^	*p*^corr^
**Frequencies (%) in γδ T cells**				
Vδ1^+^	38.80 ± 25.53	21.24 ± 18.38	0.0057	0.0513
Vδ2^+^	32.12 ± 22.88	52.95 ± 23.07	0.0033	0.0297
Vδ1^−^Vδ2^−^	27.08 ± 15.47	23.84 ± 11.92	NS	NS
Vδ1^+^Vγ9^+^	8.85 ± 11.09	3.10 ± 3.98	0.0147	NS
Vδ1^+^Vγ9^−^	29.92 ± 19.18	18.00 ± 17.50	0.0088	0.0792
Vδ2^+^Vγ9^+^	31.69 ± 22.71	52.57 ± 23.12	0.0032	0.0288
Vδ2^+^Vγ9^−^	0.30 ± 0.43	0.32 ± 0.47	NS	NS
Vδ1^−^Vδ2^−^Vγ9^+^	2.84 ± 6.20	4.60 ± 5.37	0.0098	0.0882
Vδ1^−^Vδ2^−^Vγ9^−^	24.23 ± 13.17	19.18 ± 12.29	NS	NS

**Frequencies (%) in total CD3^+^ T cells**				
Total γδ T cells	3.96 ± 3.02	4.64 ± 2.44	NS	NS
Vδ1^+^	1.71 ± 2.19	1.13 ± 1.53	NS	NS
Vδ2^+^	1.29 ± 1.52	2.47 ± 1.86	0.0038	0.0380
Vδ1^−^Vδ2^−^	0.88 ± 0.65	0.95 ± 0.54	NS	NS
Vδ1^+^Vγ9^+^	0.38 ± 0.58	0.14 ± 0.22	NS	NS
Vδ1^+^Vγ9^−^	1.33 ± 1.92	0.98 ± 1.44	NS	NS
Vδ2^+^Vγ9^+^	1.28 ± 1.52	2.45 ± 1.85	0.0034	0.0340
Vδ2^+^Vγ9^−^	0.01 ± 0.01	0.01 ± 0.03	NS	NS
Vδ1^−^Vδ2^−^Vγ9^+^	0.08 ± 0.14	0.24 ± 0.32	0.0036	0.0360
Vδ1^−^Vδ2^−^Vγ9^−^	0.80 ± 0.63	0.71 ± 0.44	NS	NS

*All data are presented as the mean ± SD. p^uncorr^ was corrected by multiplying by 9 for the frequencies in γδ T cells and by 10 for that in total CD3^+^ T cells to calculate the p^corr^*.

*HCs, healthy controls; MS, multiple sclerosis; NS, not significant*.

### Altered Cytokine Production by γδ T Cells in MS Patients

Regarding cytokine production by γδ T cells, IFN-γ^+^ cells in Vδ2^+^ γδ T cells and IL-17A^+^ cells in Vδ1^−^Vδ2^−^ γδ T cells were significantly decreased in MS patients compared with HCs (*p*^corr^ = 0.0054 and *p*^corr^ = 0.0171, respectively) (Table 3). The percentages of IL-17A^+^IFN-γ^+^ cells in Vδ2^+^ γδ T cells and IFN-γ^+^ cells in Vδ1^−^Vδ2^−^ γδ T cells also tended to be lower in MS than in HCs (*p*^corr^ = 0.0882 and *p*^corr^ = 0.0855, respectively). In total γδ T cells, IL-17A or IFN-γ production by Vδ1^+^ γδ T cells was comparable between MS patients and HCs, whereas the percentages of IFN-γ^+^Vδ2^+^ and IL-17A^+^IFN-γ^+^Vδ2^+^ γδ T cells were significantly lower in MS patients than in HCs (*p*^corr^ < 0.0005 and *p*^corr^ = 0.0135, respectively).

**Table 3:** Comparison of cytokine-producing γδ T cell subpopulations between MS patients in remission and HCs.

	MS (*n* = 30)	HCs (*n* = 23)	*p*^uncorr^	*p*^corr^
**Frequencies (%) in each γδ T cell subpopulation**				
Vδ1^+^γδ T cells				
IL-17A^+^	0.28 ± 0.61	0.25 ± 0.36	NS	NS
IFN-γ^+^	28.88 ± 19.24	44.06 ± 27.30	0.0771	NS
IL-17A^+^IFN-γ^+^	1.30 ± 6.06	0.35 ± 0.66	NS	NS
Vδ2^+γδ^ T cells				
IL-17A^+^	0.19 ± 0.44	0.22 ± 0.67	NS	NS
IFN-γ^+^	43.90 ± 32.26	75.06 ± 22.86	0.0006	0.0054
IL-17A^+^IFN-γ^+^	0.31 ± 0.76	0.57 ± 1.12	0.0098	0.0882
Vδ1^−^Vδ2^−^γδ T cells				
IL-17A^+^	0.45 ± 0.60	1.23 ± 1.00	0.0019	0.0171
IFN-γ^+^	28.31 ± 17.20	43.46 ± 18.66	0.0095	0.0855
IL-17A^+^IFN-γ^+^	0.55 ± 1.03	1.06 ± 1.42	0.0143	NS

**Frequencies (%) in total γδ T cells**				
Vδ1^+^γδ T cells				
IL-17A^+^	0.20 ± 0.50	0.09 ± 0.18	NS	NS
IFN-γ^+^	11.23 ± 10.84	11.43 ± 16.29	NS	NS
IL-17A^+^IFN-γ^+^	0.14 ± 0.50	0.06 ± 0.13	NS	NS
Vδ2^+^γδ T cells				
IL-17A^+^	0.12 ± 0.25	0.37 ± 0.67	0.0142	NS
IFN-γ^+^	13.45 ± 15.42	41.16 ± 22.82	<0.0001	<0.0005
IL-17A^+^IFN-γ^+^	0.07 ± 0.22	0.27 ± 0.42	0.0015	0.0135
Vδ1^−^Vδ2^−^γδ T cells				
IL-17A^+^	0.31 ± 0.54	0.54 ± 0.77	0.0178	NS
IFN-γ^+^	8.22 ± 7.82	10.04 ± 6.26	NS	NS
IL-17A^+^IFN-γ^+^	0.16 ± 0.31	0.26 ± 0.55	NS	NS

*All data are presented as the mean ± SD. p^uncorr^ was corrected by multiplying the value by nine to calculate the p^corr^*.

*HCs, healthy controls; IFN, interferon; IL, interleukin; MS, multiple sclerosis; NS, not significant*.

### αβ T Cell Subpopulations in MS Patients

There was no significant difference in the percentages of Tnaïve, Tcm, Tem, Teff, and activated T cells in the CD4^+^ and CD8^+^ T cell populations between MS patients in remission and HCs (Table 4). However, MS patients had a lower percentage of Treg cells in CD4^+^ T cells than HCs by uncorrected statistical analysis, although the statistical significance was lost after Bonferroni–Dunn’s correction (*p*^uncorr^ = 0.0201, *p*^corr^ = 0.2211).

**Table 4:** Comparison of αβ T cell subpopulations between MS patients in remission and HCs.

	MS (*n* = 30)	HCs (*n* = 23)	*p*^uncorr^	*p*^corr^
**CD4^+^ T cell subpopulations (%)**				
Tnaïve (CCR7^+^CD45RA^+^)	46.59 ± 15.30	44.98 ± 16.02	NS	NS
Tcm (CCR7^+^CD45RA^−^)	27.86 ± 8.51	29.06 ± 9.00	NS	NS
Tem (CCR7^−^CD45RA^−^)	22.17 ± 10.67	23.35 ± 10.99	NS	NS
Teff (CCR7^−^CD45RA^+^)	3.39 ± 1.93	2.61 ± 1.48	NS	NS
Activated T (HLA-DR^+^)	2.23 ± 1.23	3.39 ± 3.16	NS	NS
Treg (CD25^+^CD127^low/−^)	4.59 ± 1.68	5.75 ± 1.82	0.0201	NS

**CD8^+^ T cell subpopulations (%)**				
Tnaïve (CCR7^+^CD45RA^+^)	30.43 ± 21.11	39.02 ± 18.44	0.0560	NS
Tcm (CCR7^+^CD45RA^−^)	6.14 ± 3.74	10.40 ± 13.14	NS	NS
Tem (CCR7^−^CD45RA^−^)	41.60 ± 18.49	37.06 ± 19.39	NS	NS
Teff (CCR7^−^CD45RA^+^)	21.60 ± 16.92	12.78 ± 7.11	0.0571	NS
Activated T (HLA-DR^+^)	5.25 ± 2.78	4.67 ± 3.66	NS	NS

*All data are presented as the mean ± SD. p^uncorr^ was corrected by multiplying the value by 11 to calculate the p^corr^*.

*HCs, healthy controls; MS, multiple sclerosis; NS, not significant; Tnaïve, naïve T; Tcm, central memory T; Tem, effector memory T; Teff, effector T; Treg, regulatory CD4^+^ T*.

### Cytokine Production by αβ T Cells in MS Patients

The percentages of IL-17A, IFN-γ, IL-4, and GM-CSF-producing cells in CD4^+^ T cells, and IL-17A and IFN-γ-producing cells in CD8^+^ T cells were not significantly different between MS patients in remission and HCs after Bonferroni–Dunn’s correction (Table 5).

**Table 5:** Comparison of cytokine-producing αβ T cell subpopulations between MS patients in remission and HCs.

	MS (*n* = 30)	HCs (*n* = 23)	*p*^uncorr^	*p*^corr^
**Frequencies (%) in CD4^+^ T cells**				
IL-17A^+^	0.34 ± 0.25	0.75 ± 0.96	NS	NS
IFN-γ^+^	7.12 ± 6.14	9.46 ± 7.63	NS	NS
IL-4^+^	1.95 ± 1.26	3.04 ± 2.22	NS	NS
GM-CSF^+^	2.38 ± 2.24	5.31 ± 5.10	0.0756	NS
IL-17A^+^IFN-γ^+^	0.05 ± 0.07	0.11 ± 0.19	NS	NS
IL-17A^+^GM-CSF^+^	0.07 ± 0.08	0.26 ± 0.42	0.0337	NS

**Frequencies (%) in CD8^+^ T cells**				
IL-17A^+^	0.24 ± 0.21	0.22 ± 0.17	NS	NS
IFN-γ^+^	23.62 ± 16.70	27.60 ± 21.55	NS	NS
IL-17A^+^IFN-γ^+^	0.10 ± 0.12	0.09 ± 0.12	NS	NS

*All data are presented as the mean ± SD. p^uncorr^ was corrected by multiplying the value by nine to calculate the p^corr^*.

*GM-CSF, granulocyte-macrophage colony-stimulating factor; HCs, healthy controls; IFN, interferon; IL, interleukin; MS, multiple sclerosis; NS, not significant*.

### B Cell Subpopulations in MS Patients

The percentages of naïve, total memory, CS^+^ memory, transitional B cells, and plasmablasts in total B cells did not significantly differ between MS patients in remission and HCs (Table 6). However, the percentages of CS^−^ memory B cells was slightly but significantly decreased in MS patients compared with HCs (*p*^corr^ = 0.0204).

**Table 6:** Comparison of B cell subpopulations between MS patients in remission and HCs.

	MS (*n* = 30)	HCs (*n* = 23)	*p*^uncorr^	*p*^corr^
**Frequencies (%) in total B cells**				
Naïve (CD27^−^IgD^+^)	45.02 ± 16.39	49.17 ± 13.26	NS	NS
Memory (CD27^+^)	20.14 ± 13.80	21.96 ± 7.53	NS	NS
CS^+^ memory (CD27^+^IgD^−^)	17.50 ± 11.21	18.34 ± 6.10	NS	NS
CS^−^ memory (CD27^+^IgD^+^)	2.30 ± 2.05	3.62 ± 2.67	0.0034	0.0204
Plasmablasts (CD38^high^CD20^−^)	0.55 ± 0.59	0.36 ± 0.22	NS	NS
Transitional (CD24^high^CD38^high^)	3.92 ± 3.01	3.59 ± 2.18	NS	NS

*All data are presented as the mean ± SD. p^uncorr^ was corrected by multiplying the value by six to calculate the p^corr^*.

*CS, class switched; HCs, healthy controls; MS, multiple sclerosis; NS, not significant*.

### Correlation of Altered γδ T Cell Repertoires With Disease Severity in MS Patients

**Figure 2:**
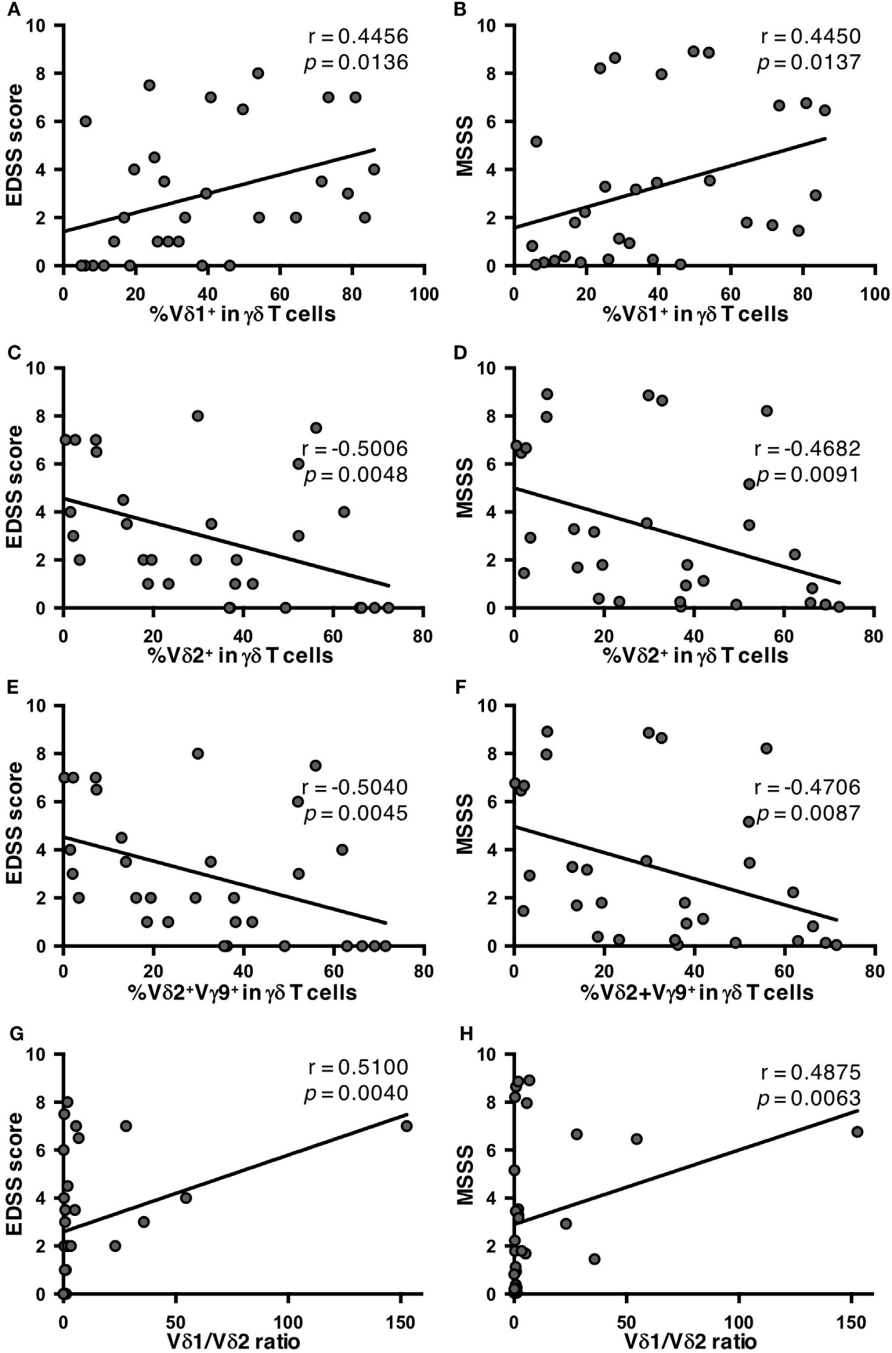
Correlations of percentages of γδ T cell subpopulations with disease severity in MS patients. **(A)** Correlation between EDSS scores and the percentage of Vδ1^+^ cells in γδ T cells. **(B)** Correlation between MSSS and the percentage of Vδ1^+^ cells in γδ T cells. **(C)** Correlation between EDSS scores and the percentage of Vδ2^+^ cells in γδ T cells. **(D)** Correlation between MSSS and the percentage of Vδ2^+^ cells in γδ T cells. **(E)** Correlation between EDSS scores and the percentage of Vδ2^+^Vγ9^+^ cells in γδ T cells. **(F)** Correlation between MSSS and the percentage of Vδ2^+^Vγ9^+^ cells in γδ T cells. **(G)** Correlation between EDSS scores and Vδ1/Vδ2 ratio. **(H)** Correlation between MSSS and Vδ1/Vδ2 ratio. Abbreviations: HCs, healthy controls; EDSS, Expanded Disability Status Scale; MS, multiple sclerosis; MSSS, Multiple Sclerosis Severity Score.

To elucidate the clinical relevance of an altered γδ T cell repertoire in MS, potential correlations of γδ T cell subpopulations with the clinical demographics of MS patients were analyzed. There was no significant correlation of any γδ T cell parameter with age at disease onset, disease duration, or annualized relapse rates (data not shown). However, the percentages of Vδ2^+^ and Vδ2^+^Vγ9^+^ cells in γδ T cells had significant negative correlations with Expanded Disability Status Scale (EDSS) scores (Vδ2^+^: *r* = −0.5006, *p* = 0.0048; and Vδ2^+^Vγ9^+^: *r* = −0.5040, *p* = 0.0045), and Multiple Sclerosis Severity Score (MSSS) (Vδ2^+^: *r* = −0.4682, *p* = 0.0091; and Vδ2^+^Vγ9^+^: *r* = −0.4706, *p* = 0.0087). By contrast, the percentage of Vδ1^+^ cells in γδ T cells had a significant positive correlation with these parameters (EDSS score: *r* = 0.4456, *p* = 0.0136; and MSSS: *r* = 0.4450, *p* = 0.0137) (Figures 2A–F). As a result, the Vδ1/Vδ2 ratio was positively correlated with both EDSS scores and MSSS (*r* = 0.5100, *p* = 0.0040; and *r* = 0.4875, *p* = 0.0063, respectively) (Figures 2G,H). Subsequently, the correlation of cytokine-producing γδ T cells and clinical parameters in MS patients were also analyzed. The percentages of IL-17^+^, IFN-γ^+^, or IL-17A^+^IFN-γ^+^ cells in Vδ2^+^ or total γδ T cells did not correlate with age at disease onset, disease duration, annualized relapse rate, EDSS score or MSSS in MS patients (data not shown). These findings were reproduced even when patients were limited to relapsing-remitting MS cases (data not shown).

### Correlations of Altered γδ T Cell Repertoires With Regulatory T Cells in MS Patients

**Figure 3:**
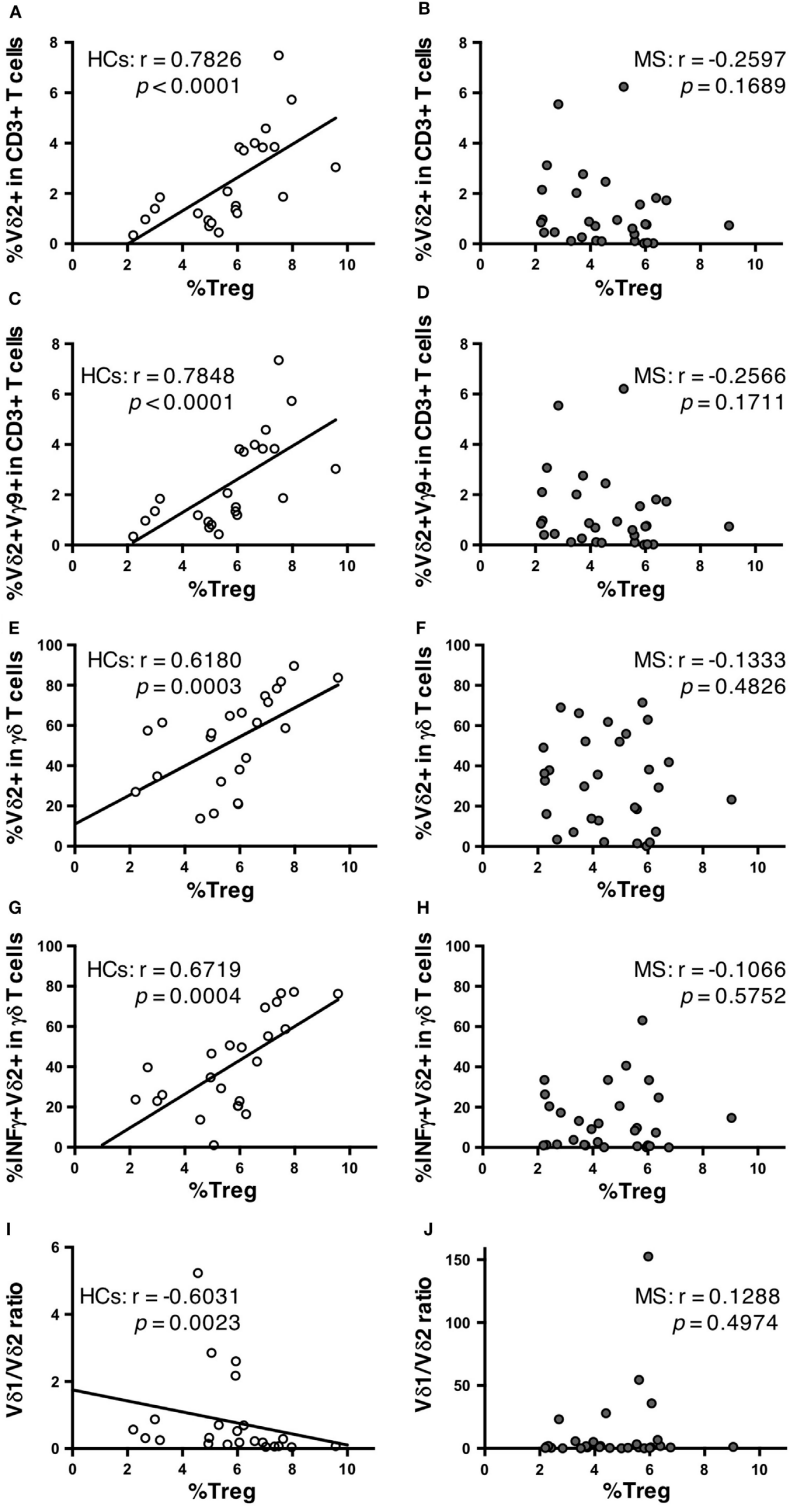
Correlations between γδ T cell subpopulations and Treg cells in HCs and MS patients. Correlation between the percentages of Treg cells among CD4^+^ T cells and Vδ2^+^ cells among CD3^+^ T cells in HCs **(A)** and MS patients **(B)**. Correlation between the percentages of Treg cells among CD4^+^ T cells and Vδ2^+^Vγ9^+^ cells among CD3^+^ T cells in HCs **(C)** and MS patients **(D)**. Correlation between the percentages of Treg cells among CD4^+^ T cells and Vδ2^+^ cells among γδ T cells in HCs **(E)** and MS patients **(F)**. Correlation between the percentage of Treg cells among CD4^+^ T cells and IFN-γ^+^Vδ2^+^ among γδ T cells in HCs **(G)** and MS patients **(H)**. Correlations between the percentage of Treg cells among CD4^+^ T cells and Vδ1/Vδ2 ratio in HCs **(I)** and MS patients **(J)**. Abbreviations: HCs, healthy controls; MS, multiple sclerosis; Treg, regulatory CD4^+^ T; IFN, interferon.

Finally, potential correlations of γδ T cell repertoires with αβ T cell and B cell subpopulations were also analyzed in MS patients and HCs. No significant association of γδ T cell subpopulations with any αβ T cell or B cell subpopulation was found in MS patients or HC (data not shown), except for Treg cells. Surprisingly, in HCs, the percentages of Vδ2^+^ cells and Vδ2^+^Vγ9^+^ cells in total CD3^+^ T cells had highly significant positive correlations with Treg cell percentages in CD4^+^ T cells (*r* = 0.7826, *p* < 0.0001; and *r* = 0.7848, *p* < 0.0001, respectively) (Figures 3A,C). The same was also observed for γδ T cell percentages in total CD3^+^ T cells (*r* = 0.4829, *p* = 0.0196). The percentages of Vδ2^+^, Vδ2^+^Vγ9^+^, and IFN-γ^+^Vδ2^+^ cells in γδ T cells also showed significant positive correlations with Treg cell percentages in CD4^+^ T cells (*r* = 0.6810, *p* = 0.0003; *r* = 0.6868, *p* = 0.0003; and *r* = 0.6719, *p* = 0.0004, respectively) (Figures 3E,G). The percentages of Vδ1^+^ in γδ T cells and the Vδ1/Vδ2 ratio had significant negative correlations with Treg cell percentages in CD4^+^ T cells (*r* = −0.5504, *p* = 0.0065; and *r* = −0.6031, *p* = 0.0023, respectively) (Figure 3I). By contrast, such correlations were totally lost in MS patients (Figures 3B,D,F,H,J).

## Discussion

The present study is the first to report the following characteristic features of γδ T cells in MS: (1) a decrease of Vδ2^+^, Vδ2^+^Vγ9^+^ cells, and IFN-γ^+^Vδ2^+^ γδ T cells; (2) negative correlations between the percentages of Vδ2^+^Vγ9^+^ cells in γδ T cells and disease severity as determined by EDSS scores and MSSS; and (3) lack of positive correlations between the percentages of Vδ2^+^, Vδ2^+^Vγ9^+^, and IFN-γ^+^Vδ2^+^ cells in γδ T cells and of CD3^+^ T cells with Treg cell percentages in CD4^+^ T cells in HCs.

Two previous studies reported an increase of Vδ1^+^ T cells in MS (21, 22), which is partly in accord with the increased Vδ1/Vδ2 ratios evident in our MS cohort. However, neither study specified the disease phase of the MS patients nor examined the relationship of γδ T cells with other immune cell populations measured simultaneously. In the two previous studies, Zeine et al. (21) regarded Vγ9^+^TCRγδ^+^CD3^+^ cells as Vδ2^+^ γδ T cells, while Singh et al. (22) counted all Vδ1^−^ cells as Vδ2^+^ γδ T cells, instead of directly staining Vδ2. Therefore, these two studies might have overestimated Vδ2^+^ γδ T cells according to our data shown in Table 2. We considered a decrease of Vδ2^+^ T cells to be a primary change, because the decrease of Vδ2^+^ T cells but not increase of Vδ1^+^ T cells was significant even after Bonferroni–Dunn’s correction to minimize statistical error.

Vδ2^+^Vγ9^+^ cells, which are the majority of γδ T cells in human peripheral blood (5), recognize phosphorylated antigens of microbes, such as mycobacterium, and function in host defense by producing proinflammatory cytokines, including tumor necrosis factor-α, IL-17, and IFN-γ (5, 9, 10). There is also increasing evidence for the regulatory functions of γδ T cells. Vδ2^+^ γδ T cells were reported to express FoxP3 and regulate the proliferation of αβ T cells (30), producing anti-inflammatory cytokines, such as transforming growth factor-β (31, 32). Furthermore, IFN-γ-producing γδ T cells suppressed Th17 cell responses in murine pulmonary fibrosis models (33). Therefore, a decrease of Vδ2^+^ γδ T cells, especially Vδ2^+^Vγ9^+^ γδ T cells, in our MS patients might contribute to disease severity, possibly in part through reducing the regulatory functions against autoreactive αβ T cells. Although the percentages of Vδ2^+^ and Vδ2^+^Vγ9^+^ cells in γδ T cells had strong negative correlations with EDSS scores and MSSS, IFN-γ^+^Vδ2^+^ γδ T cells did not. Therefore, cytokines other than IFN-γ might also be critically involved in the reduction of disease activity by Vδ2^+^Vγ9^+^ cells. Alternatively, Vδ2^+^Vγ9^+^ γδ T cells might migrate into the CNS of MS patients, resulting in their decreased number in the peripheral blood. However, because all patients in the present study were stable and in the remission phase without receiving DMTs, disease activity-related invasion of these cells into the CNS seems unlikely.

Intriguingly, a strong positive correlation of the percentages of Vδ2^+^, Vδ2^+^Vγ9^+^, and IFN-γ^+^Vδ2^+^ cells in CD3^+^ T cells (and in γδ T cells) with Treg percentages in CD4^+^ T cells was found in HCs, whereas such a correlation was completely missing in our untreated MS patients. In MS, Treg cell dysfunction has been repeatedly reported (34–36); however, the mechanism by which Treg cell numbers are normally controlled and how regulatory function is impaired in MS are still ill defined. Thus, to the best of our knowledge, our study is the first to demonstrate a close association between Treg cell populations and a specific γδ T cell subset, i.e., Vδ2^+^ γδ T cells, in healthy humans. Although such a correlation has not been established in experimental animals, it was reported that a murine autoimmune keratitis model using TCRδ knockout mice had few peripheral blood CD4^+^CD25^+^Foxp3^+^ Treg cells and highly activated memory CD8^+^ T cells (37). It is possible that the normal regulatory functions of Vδ2^+^ γδ T cells on Treg cells are lost in MS, although the underlying mechanism remains to be elucidated. Indeed, Treg cell percentages were significantly reduced in our MS patients compared with HCs before Bonferroni–Dunn’s correction. Several studies have reported a mild decrease of CD4^+^CD25^high^ Treg cell numbers, impaired suppressive functions of Treg cells, and improvement of Treg cell functions by IFN-β treatment in MS (34–36). Therefore, we consider the relationship between the actual regulatory functions of CD4^+^ Treg cells and the frequency of Vδ2^+^, Vδ2^+^Vγ9^+^, and IFN-γ-producing Vδ2^+^ γδ T cells worth investigating in future MS studies.

Another major γδ T cell subset is Vδ1^+^ γδ T cells that recognize self-lipid antigens presented by CD1d molecules on antigen-presenting cells, such as dendritic cells (38). Recently, several studies revealed that Vδ1^+^ γδ T cells reacted to sulfatide, a glycosphingolipid antigen abundantly present in the myelin sheath (39–41). Thus, the increase of Vδ1^+^ γδ T cells in peripheral blood from MS patients from different races observed by two previous studies (21, 22) and the present study may account for the prominence of Vδ1^+^ γδ T cells in MS lesions. Collectively, the decrease of Vδ2^+^ γδ T cells may dampen the immunoregulatory functions of γδ T cells in peripheral blood, while the increase of Vδ1^+^ γδ T cells may enhance tissue damage in MS lesions in the CNS. As a result, the Vδ1/Vδ2 ratio is closely associated with disease severity in MS, suggesting that the Vδ1/Vδ2 ratio might be a candidate biomarker for predicting disease severity in MS.

There were several limitations to the present study. First, the sample size was relatively small due to the rarity of MS in Asians (42) and the enrollment of MS patients not on DMTs. Therefore, we analyzed our data using Bonferroni–Dunn’s correction for multiple comparisons to minimize errors derived from sample size. Second, a comparison of the immunoprofile between patients in the relapse and remission phases was lacking. Thus, our results should be confirmed by studies using larger numbers of MS patients in both relapse and remission phases in the future. Third, we did not measure the suppressive activity of the CD4^+^CD25^+^CD127^low/−^ T cells defined as Treg cells in the present study. Instead, we measured expression of FoxP3 in CD4^+^CD25^+^CD127^low/−^ T cells and confirmed that most CD4^+^CD25^+^CD127^low/−^ T cells expressed FoxP3. Moreover, the percentages of CD4^+^CD25^+^CD127^low/−^ T cells had a significant positive correlation with those of CD4^+^CD25^+^FoxP3^+^ T cells in CD4^+^ T cells. Although FoxP3^+^CD4^+^CD25^+^ T cells have been reported to exert the suppressive functions of Treg cells (26, 27), we consider it necessary to measure the suppressive functions of CD4^+^CD25^+^CD127^low/−^ T cells in future. Thus, the positive correlation between the percentages of Vδ2^+^ cells and Vδ2^+^Vγ9^+^ cells in total CD3^+^ T cells and Treg cell percentages in CD4^+^ T cells should be regarded as preliminary. Fourth, we did not measure the suppressive activity of γδ T cells on αβ T cells. Functional assays of γδ T cells should be performed in a future study to clarify the interaction between Vδ2^+^ γδ T cells and Treg cells. Finally, it has repeatedly been reported that MS phenotypes differ among races. Although an increase of Vδ1^+^ γδ T cell numbers is commonly found in Caucasian and Japanese MS populations, our findings regarding Vδ2^+^ γδ T cells should be confirmed in other races.

In conclusion, our study suggests that untreated MS patients have alterations in γδ T cells even in the remission phase. Specifically, decreased numbers of Vδ2^+^Vγ9^+^ and IFN-γ^+^Vδ2^+^ cells and a relative increase of Vδ1^+^ cells may, respectively, contribute to MS severity. We propose that the Vδ1/Vδ2 ratio may be a novel biomarker for disease severity in MS.

## Ethics Statement

This present study was approved by the Ethical Committee of Kyushu University and conducted with written informed consent from all participants according to the World Medical Association Declaration of Helsinki.

## Author Contributions

KS, GM, and JK conceived and designed the study. KS, YN, KM, TM, NI, RY, and JK collected data. YY contributed to the design and implementation of the experiments. KS and GM performed experiments and analyzed data. GM, KS, and JK wrote the paper.

## Conflict of Interest Statement

GM, YN, and KM have nothing to declare. KS has received speaking honoraria from Takeda Pharmaceutical Company and Biogen Idec Japan. TM has received grants and payments from Bayer Schering Pharma, Takeda Pharmaceutical Company, Mitsubishi Tanabe Pharma, Bayer Schering Pharma, and Biogen Idec Japan. RY has received research support from Bayer Schering Pharma, Biogen Idec Japan, Novartis Pharma, and Mitsubishi Tanabe Pharma. JK is a consultant for Biogen Idec Japan and Medical Review. He has received honoraria from Bayer Healthcare, Mitsubishi Tanabe Pharma, Nobelpharma, Otsuka Pharmaceutical, and Medical Review.

## Abbreviations

CNS, central nervous system; CNV, copy number variation; EDSS, Expanded Disability Status Scale; HCs, healthy controls; IFN, interferon; IL, interleukin; MS, multiple sclerosis; TCR, T cell receptor; Tcm, central memory T; Teff, effector T; Tem, effector memory T; Th, helper T; Treg, regulatory CD4^+^ T.
