# Supplementary material for: Association of Decreased Percentage of Vδ2+Vγ9+ γδ T Cells With Disease Severity in Multiple Sclerosis
Source: Front Immunol. 2018 Apr 10;9:748. doi: 10.3389/fimmu.2018.00748 (PMC5903009; doi:10.3389/fimmu.2018.00748)
Supplement: Supplementary file 1 [file table_1.docx]

**Table S1. Sources of antibodies and reagents used in the study.**

| **Vender** | **Conjugated antibodies** | **Clone** |
| --- | --- | --- |
| BD Biosciences (San Jose, CA) | CD3–PerCP-Cy5.5 | UCHT1 |
|  | CD3ε–APC-H7 | SK-7 |
|  | CD8–PE-Cy7 | RPA-T8 |
|  | CD14–V500 | M5E2 |
|  | CD19–V500 | HIB19 |
|  | CD20–APC-H7 | 2H7 |
|  | CD27–PE-Cy7 | M-T271 |
|  | CD45RA–APC-H7 | HI100 |
|  | CD127–APC | HIL-7R-M21 |
|  | CCR7(CD197)–PE | 150503 |
|  | HLA-DR–V500 | G46-6 |
|  | IgD–V500 | IA6-2 |
|  | IL-17A–BV421 | N49-653 |
|  | TCR Vδ2–PE | B6 |
|  | TCR Vγ9–APC | B3 |
|  | TCR γδ­–BV421 | B1 |
| Biolegend (San Diego, CA) | CD4–FITC | RPA-T4 |
|  | CD4–Biotin | RPA-T4 |
|  | CD4–PE | RPA-T4 |
|  | CD19–FITC | HIB19 |
|  | CD24–PE | ML5 |
|  | CD25–BV421 | BC96 |
|  | CD38–APC | HB-7 |
|  | IFN-γ–PerCP-Cy5.5 | 4S.B3 |
|  | IL-4–PE | MP4-25D2 |
|  | GM-CSF–APC | BVD-21C11 |
| eBioscience (San Diego, CA) | Foxp3–Alexa488 | PCH101 |
|  | IL-17A–eFlour 660 | 64DEC17 |
|  | TCR αβ–PE-Cy7 | IP-26 |
| Thermo Fisher Scientific (Waltham, MA) | TCR Vδ1–FITC | TS-1 |
| Invitrogen (Carlsbad, CA) | Streptavidin-Alexa Fluor 488 |  |
