# Supplementary material for: Association of Decreased Percentage of Vδ2+Vγ9+ γδ T Cells With Disease Severity in Multiple Sclerosis
Source: Front Immunol. 2018 Apr 10;9:748. doi: 10.3389/fimmu.2018.00748 (PMC5903009; doi:10.3389/fimmu.2018.00748)
Supplement: Supplementary file 2 [file table_2.docx]

**Table S2. Panels of antibodies used for immunophenotyping T and B lymphocytes.**

| **Fluorochrome** | **Surface 1**  **αβ T cells** | **Surface 2**  **γδ T cells** | **Surface 3**  **B cells** | **Cytokines 1**  **αβ T cells** | **Cytokines 2**  **γδ T cells** | **Foxp3**  **Treg cells** |
| --- | --- | --- | --- | --- | --- | --- |
| FITC | CD4 | Vδ1 | CD19 | CD4 | Vδ1 | Foxp3 |
| PE | CCR7 | Vδ2 | CD24 | IL-4 | Vδ2 | CD4 |
| PerCP-Cy5.5 | CD3 | – | CD3 | IFN-γ | IFN-γ | – |
| PE-Cy7 | CD8 | TCRαβ | CD27 | CD8 | TCRαβ | CD8 |
| APC | CD127 | Vγ9 | CD38 | GM-CSF | IL-17A | CD127 |
| APC-H7 | CD45RA | CD3e | CD20 | CD3 | CD3e | CD3 |
| BV421 | CD25 | TCRγδ | – | IL-17A | TCRγδ | CD25 |
| V500 | HLA-DR | CD14,CD19 | IgD | CD14,CD19 | CD14,CD19 | CD14,CD19 |

Abbreviations: APC, allophycocyanin; FITC, fluorescein isothiocyanate conjugate; GM-CSF, granulocyte-macrophage colony stimulating factor; IFN, interferon; IL, interleukin; PerCP, peridinin-chlorophyll-protein complex; TCR, T cell receptor; Treg, regulatory CD4^+^ T cells.
