# Supplementary material for: Association of Decreased Percentage of Vδ2+Vγ9+ γδ T Cells With Disease Severity in Multiple Sclerosis
Source: Front Immunol. 2018 Apr 10;9:748. doi: 10.3389/fimmu.2018.00748 (PMC5903009; doi:10.3389/fimmu.2018.00748)
Supplement: Supplementary file 3 [file image_1.PDF]

Supplementary Materials

Association of decreased percentage of Vδ2$^+$Vγ9$^+$ γδ T cells with disease severity in multiple sclerosis

Guzailiayi Maimaitijiang[1†], Koji Shinoda[1†], Yuri Nakamura[1], Katsuhisa Masaki[1], Takuya Matsushita[1], Noriko Isobe[1], Ryo Yamasaki[1], Yasunobu Yoshikai[2], Jun-ichi Kira[1*]
*Correspondence: **Prof. Jun-ichi Kira**: E-mail: kira@neuro.med.kyushu-u.ac.jp

Supplementary Figures and Tables

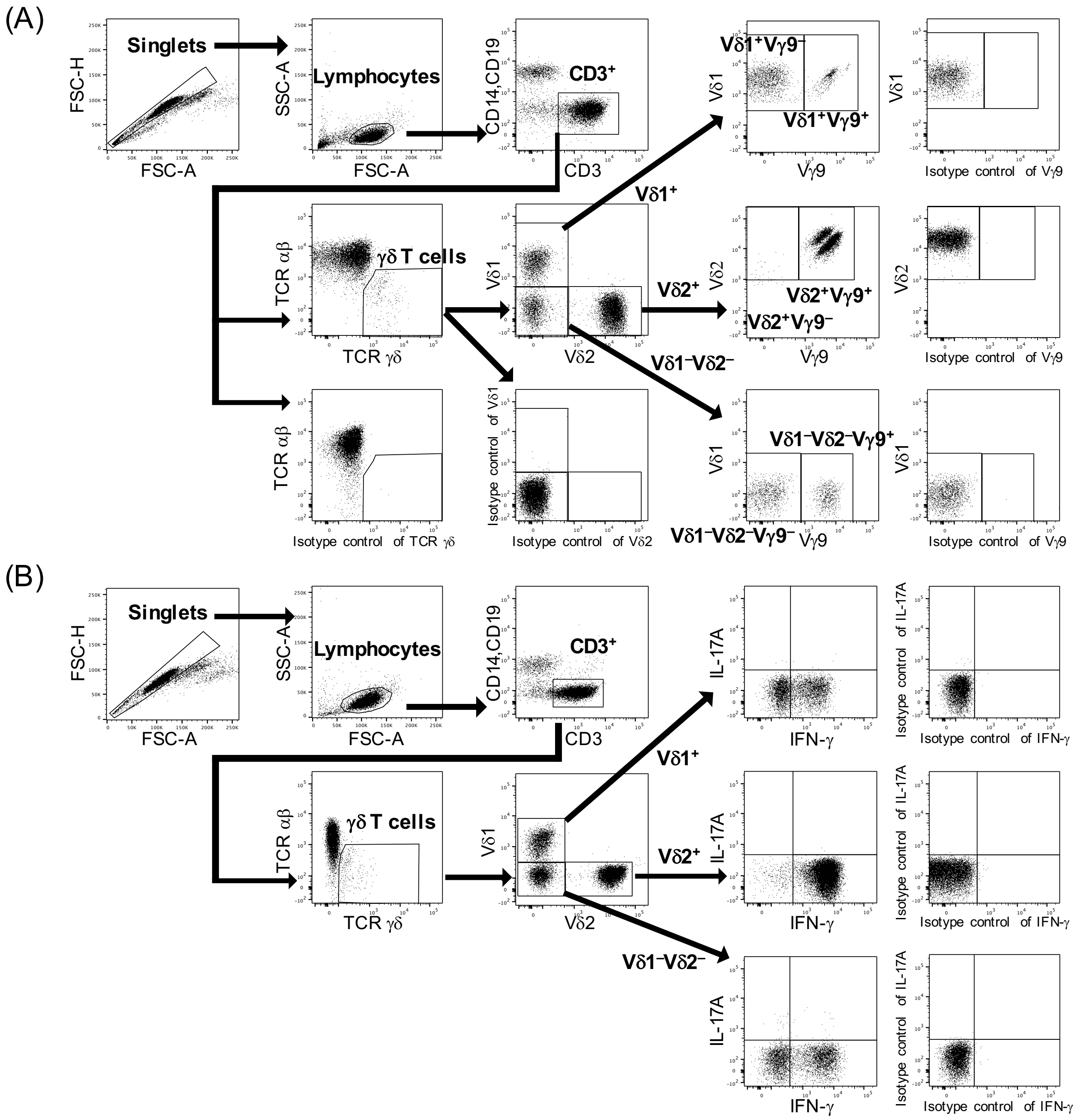

Figure S1: Immunophenotyping gating strategy for γδ T cells. Gating strategies for flow cytometric analysis in a healthy subject are shown as an example. (**A**) For the γδ T cell repertoire, peripheral blood mononuclear cells (PBMCs) were initially gated on singlet cells by forward-scatter (FSC)-area (FSC-A) and FSC-height (FSC-H), then by FSC-A and side-scatter (SSC)-area (SSC-A). γδ T cells were gated from CD3$^+$ lymphocytes by TCRγδ and TCRαβ, and classified by the presence of Vδ1 and Vδ2 into Vδ1$^+$Vδ2$^-$, Vδ1$^-$Vδ2$^+$ and Vδ1$^-$Vδ2$^-$ γδ T cells, which were then examined for the expression of Vγ9. (**B**) For intracellular cytokine staining, γδ T cells were gated from CD3$^+$ lymphocytes as TCRγδ$^+$TCRαβ$^-$ and classified according to the presence of Vδ1 or Vδ2. The cytokine production of IFN-γ and IL-17A was examined in Vδ1$^+$, Vδ2$^+$ or Vδ1$^-$Vδ2$^-$ γδ T cell populations.

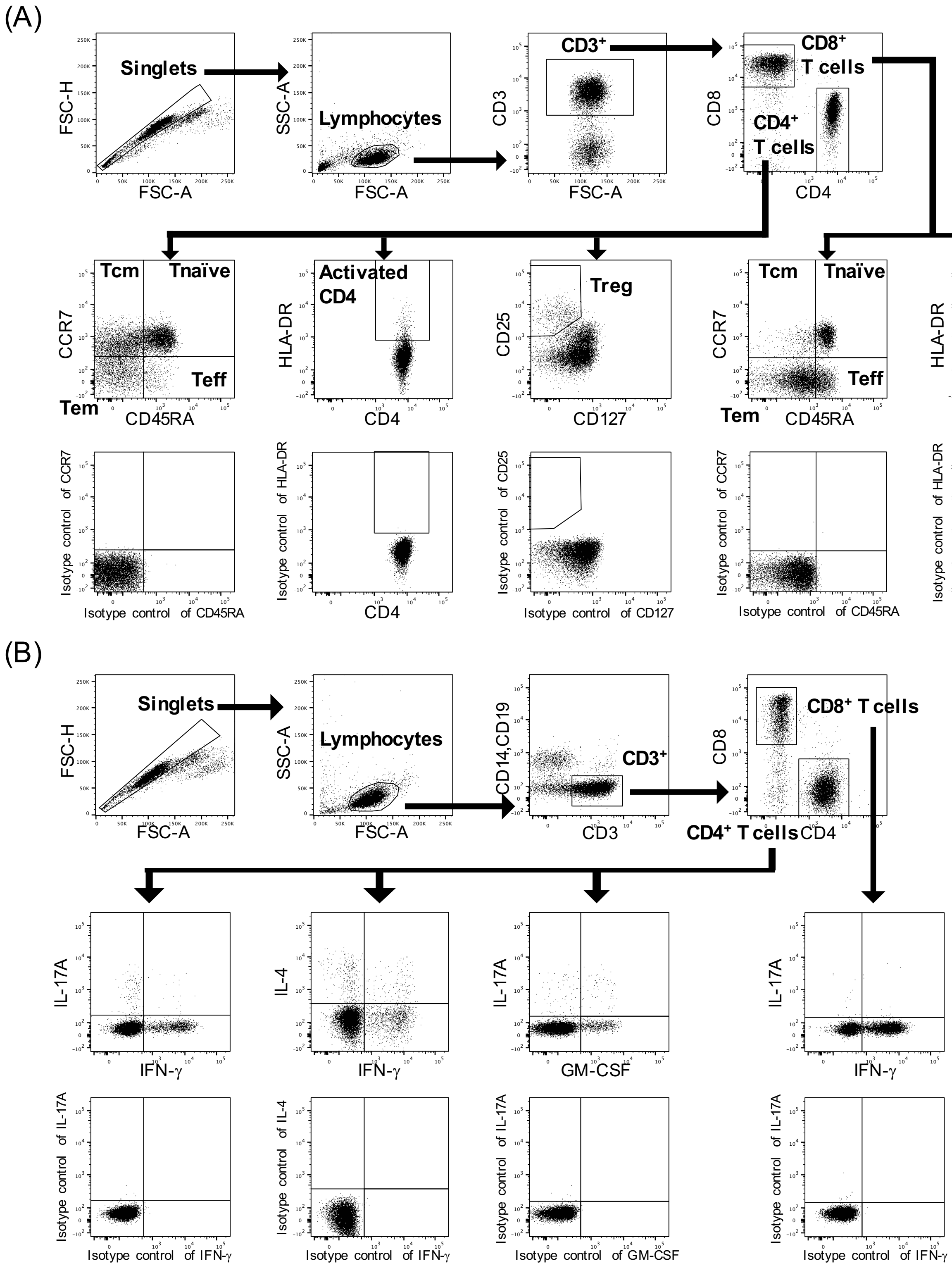

Figure S2: Immunophenotyping gating strategy for αβ T cells. Gating strategies for flow cytometric analysis in a healthy subject are shown as an example. (**A**) For the surface staining of αβ T cells, PBMCs were initially gated on singlet cells by forward-scatter (FSC)-area (FSC-A) and FSC-height (FSC-H), then by FSC-A and side-scatter (SSC)-area (SSC-A). $CD3^+$ lymphocytes were classified into $CD4^+$ and $CD8^+$ T cells. Naïve T (Tnaive) cells were defined as $CCR7^+CD45RA^+$; central memory T (Tcm) cells as $CCR7^+CD45RA^-$, effector memory T (Tem) cells as $CCR7^-CD45RA^-$, effector T (Teff) cells as $CCR7^-CD45RA^+$, activated T cells as $HLA\text{-}DR^+$ and regulatory $CD4^+$ T (Treg) cells as $CD25^+CD127^{low/-}$. (**B**) For the intracellular cytokine staining of αβ T cells, $CD3^+$ T lymphocytes were first classified as $CD4^+$ or $CD8^+$ T cells. The production of IFN-γ, IL-4, IL-17A and GM-CSF was examined in $CD4^+$ T cells, and IL-17A and IFN-γ was also measured in $CD8^+$ T cells.

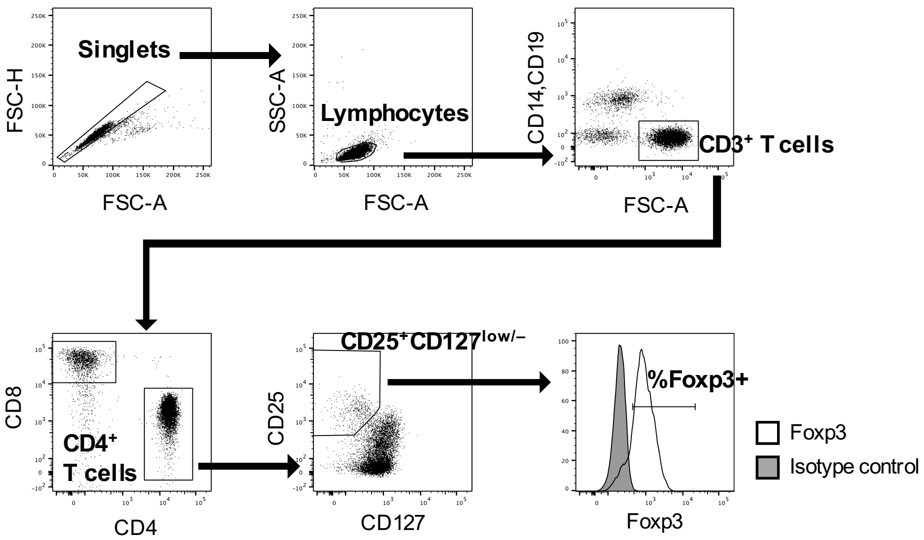

Figure S3: Immunophenotyping gating strategy for regulatory CD4$^+$ T cells. Gating strategies for flow cytometric analysis in a healthy subject are shown as an example. PBMCs were initially gated on singlet cells by forward-scatter (FSC)-area (FSC-A) and FSC-height (FSC-H), then by FSC-A and side-scatter (SSC)-area (SSC-A). CD3$^+$ lymphocytes were classified into CD4$^+$ and CD8$^+$ T cells. The expression of Foxp3 in CD25$^+$CD127$^{low/-}$ CD4$^+$ T cells were shown based on isotype matched control antibody with less than 1% background.

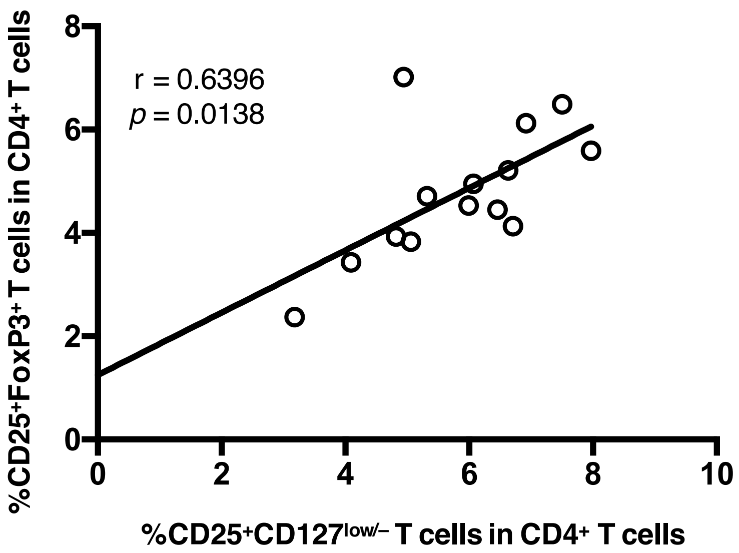

Figure S4: The correlation of percentages of $CD25^{+}CD127^{low/-}$ T cells with $CD25^{+}FoxP3^{+}$ T cells in $CD4^{+}$ T cells.

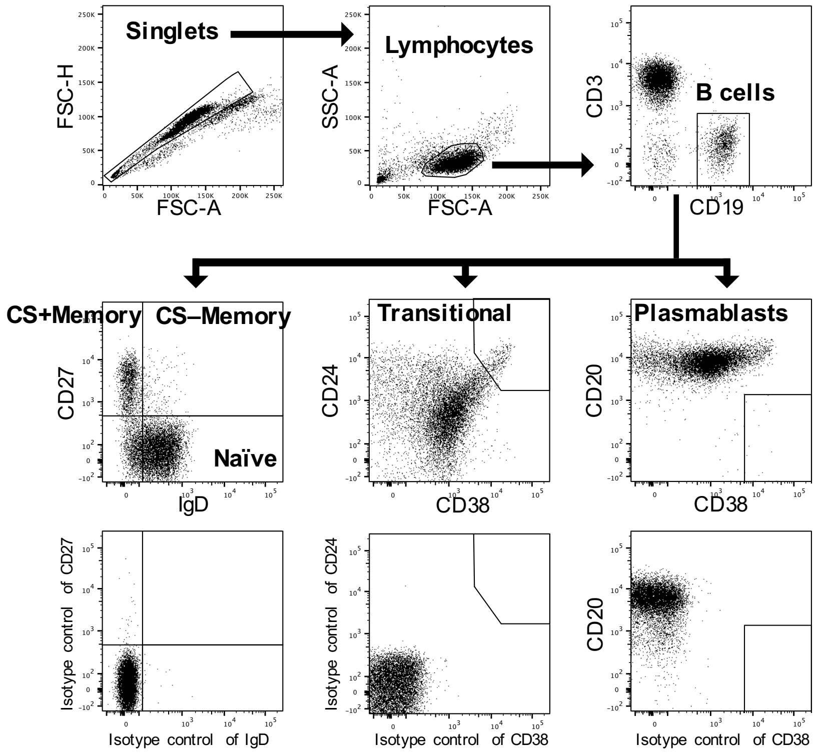

Figure S5: Immunophenotyping gating strategy for B lymphocytes. Gating strategies for flow cytometric analysis in a healthy subject are shown as an example. B lymphocytes were gated as $CD19^{+}CD3^{-}$ cells. Naïve B cells were identified as $CD27^{-}IgD^{+}$, memory B cells as $CD27^{+}$, class-switched memory B (CS+Memory) cells as $CD27^{+}IgD^{-}$, not-class-switched B (CS–Memory) cells as $CD27^{+}IgD^{+}$, transitional B cells as $CD24^{high}CD38^{high}$ and plasmablasts as $CD38^{high}CD20^{-}$.
